# Oral dydrogesterone versus oral micronized progesterone in threatened miscarriage: protocol paper for a randomized controlled trial

**DOI:** 10.1530/RAF-24-0044

**Published:** 2025-02-03

**Authors:** Alka Kriplani, Gouri Shankar Kamilya, T Ramani Devi, Ashima Taneja, Amol Pawar, Gayathri Karthik Nagesh, Tapan Pattanaik, Tanusree Gupta, Mahima Jain, Monjori Mitra

**Affiliations:** ^1^ Department of Obstetrics and Gynecology, Paras Hospitals, Gurugram, Haryana, India; ^2^ Department of Obstetrics and Gynecology, IPGME&R and SSKM Hospital, Kolkata, West Bengal, India; ^3^ Department of Obstetrics and Gynecology, Ramakrishna Medical Centre LLP, Tiruchirappalli, Tamil Nadu, India; ^4^ Department of Obstetrics and Gynecology, Dayanand Medical College & Hospital, Ludhiana, Punjab, India; ^5^ Department of Obstetrics and Gynecology, Nowrosjee Wadia Maternity Hospital, Mumbai, Maharashtra, India; ^6^ Department of Obstetrics and Gynecology, Manipal Hospital, Bengaluru, Karnataka, India; ^7^ Department of Obstetrics and Gynecology, Sum Ultimate Medicare Hospital, Bhubaneswar, Odisha, India; ^8^ Department of Gynecology and Obstetrics, Udyan Health Care Pvt. Ltd, Lucknow, Uttar Pradesh, India; ^9^ Department of Obstetrics and Gynecology, B. J. Medical College & Civil Hospital, Ahmedabad, Gujarat, India; ^10^ Department of Pediatrics, Institute of Child Health, Kolkata, West Bengal, India

**Keywords:** threatened miscarriage, oral dydrogesterone, oral micronized progesterone, pregnancy complication, randomized controlled trial

## Abstract

**Graphical abstract:**

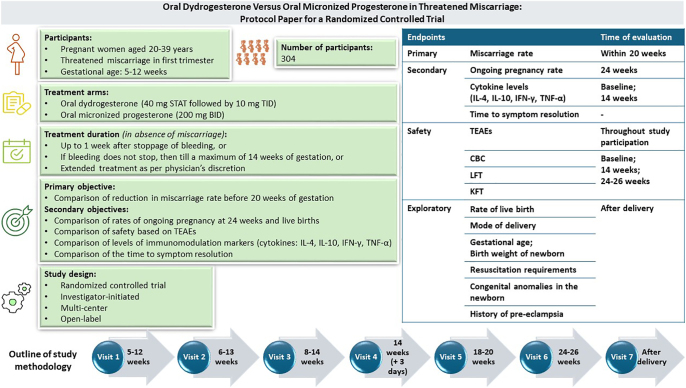

**Abstract:**

Threatened miscarriage is a common complication of early pregnancy characterized by symptoms of vaginal bleeding with/without abdominal cramps/pain in the first trimester. Progestogens are often administered for the management of this condition. Presented herein is the protocol of an ongoing, multicentric clinical trial to investigate the efficacy and safety of micronized progesterone (natural progestogen) compared to dydrogesterone (synthetic isomer of progesterone). A total of 304 eligible pregnant women aged 20–39 years, diagnosed with threatened miscarriage, will be enrolled during 5–12 weeks of gestation and randomized equally to receive either oral dydrogesterone (40 mg stat, followed by 10 mg three times a day) or oral micronized progesterone (200 mg two times a day) up to one week after stoppage of bleeding or if bleeding does not stop, then treatment will be continued till a maximum of 14 weeks of gestation (unless miscarriage is confirmed earlier or the investigator decides to prolong treatment for better outcome or if bleeding relapses). Scheduled visits after enrollment will be conducted during 6–13, 8–14, 18–20 and 24–26 weeks of gestation, in addition to a visit at the end of treatment at 14 weeks and another after parturition. The primary endpoint of the study is the miscarriage rate before 20 weeks of gestation. Secondary endpoints include the ongoing pregnancy rate at 24 weeks, treatment-induced changes in serum levels of cytokines and time to symptom resolution. Apart from the incidence of treatment-emergent adverse events, safety endpoints include changes in complete blood count and the results of liver and kidney function tests from baseline to 14 and 24–26 weeks of gestation. Delivery outcomes are exploratory endpoints of the study.

**Lay summary:**

Almost one out of four women face miscarriage during the first trimester of pregnancy; initial symptoms include vaginal bleeding with/without abdominal cramps/pain. This paper presents the plan of how an ongoing, multicentric study will be conducted to compare the efficacy and safety of oral medications known to reduce chances of miscarriage: micronized progesterone (which is a natural female sex hormone) versus synthetic progesterone. Women aged 20–39 years who are at risk of miscarriage during the first trimester of pregnancy will be randomly treated with either medication till one week after stoppage of bleeding during early pregnancy. If bleeding does not stop, treatment will be continued till a maximum of 14 weeks of pregnancy (unless miscarriage is confirmed earlier). The participants will be monitored until delivery. The study will evaluate the proportion of participants who experience miscarriage before 20 weeks of pregnancy and those who have an ongoing pregnancy at 24 weeks. It will also look at the time taken for relief from symptoms such as vaginal bleeding and abdominal pain, outcomes of delivery and incidence of any untoward event. In addition to routine tests and scans, additional tests will check for levels of biochemical parameters in the body, which are regulated by the natural or synthetic progesterone.

**Clinical trial registration number:**

CTRI/2024/02/063174 [Registered on: 26/02/2024].

## Introduction

Threatened miscarriage, a common complication of early pregnancy, is defined as ‘bleeding before 20 weeks of gestation in the presence of an embryo with cardiac activity and a closed cervix’. Vaginal bleeding is often accompanied by abdominal cramps/pain. Nearly ∼25% of women experience threatened miscarriage with the rate of early pregnancy loss being ∼11% after a live fetus is detected on ultrasonography (USG) (Hendriks *et al.* 2019). Threatened miscarriage may be associated with complications later in pregnancy. Bed rest is recommended for preventing miscarriage, but there is a dearth of convincing evidence supporting its effectiveness (Qureshi 2009). Insufficiency of progesterone during the luteal phase of menstrual cycle and early pregnancy is one of the hypothesized causes of miscarriage. Therefore, luteal support is used as a management strategy (Verma & Yadav 2021) and is achieved by the administration of progestogens (such as progesterone and dydrogesterone) or human chorionic gonadotrophin (hCG) (Qureshi 2009). Results from different clinical trials and meta-analyses have shown that progestogens may be beneficial to women with previous miscarriages and that their effects in women with no previous miscarriages remain uncertain (Coomarasamy *et al.* 2020, Devall *et al.* 2021, Zhao *et al.* 2024).

Progesterone, produced by the corpus luteum and later by the placenta, plays essential endocrinologic functions in the maintenance of pregnancy (Verma & Yadav 2021). It stimulates the growth of blood vessels supplying the endometrium, stimulates the endometrium to secrete nutrients to nurture the newly formed embryo, prepares the uterine lining for implantation and sustains the endometrium throughout pregnancy. It also inhibits inflammatory reactions by downregulating cytokine production and immune cell activation (Raghupathy & Szekeres-Bartho 2022). When used for treatment, progesterone is usually administered in a micronized form as it has very poor pharmacokinetics when taken orally (Verma & Yadav 2021). The STOP (McLindon *et al.* 2023) and PRISM (Coomarasamy *et al.* 2019) trials did not observe a significant advantage of vaginal micronized progesterone in improving live birth rate over placebo.

Dydrogesterone (6-dehydro-9β, 10α-progesterone) is similar in molecular structure and pharmacological effects to endogenous progesterone, but is more potent and has higher absorption and bioavailability than the natural progesterone due to a comparatively higher affinity for the progesterone receptor. Some studies have also highlighted the therapeutically beneficial immunomodulatory functions of the molecule (Hudić *et al.* 2011, Ibrahim *et al.* 2020). Dydrogesterone significantly decreases the levels of Th1 (proinflammatory) cytokines (such as interferon (IFN)-γ and tumor necrosis factor (TNF)-α) while significantly elevating the levels of Th2 (anti-inflammatory) cytokines (such as interleukin (IL)-4, IL-6 and IL-10). The overall effect is a significant reduction in the Th1/Th2 ratio, indicating a shift in the maternal cytokine balance from proinflammatory toward anti-inflammatory bias. This ability of dydrogesterone to downregulate proinflammatory cytokines is conducive to the continuation of a healthy pregnancy as these could otherwise be detrimental during gestation (Raghupathy & Szekeres-Bartho 2022). A significant advantage of dydrogesterone is that it retains its immunomodulatory activity even after it is converted to its major metabolite when consumed orally. In addition, it does not inhibit ovulation at recommended doses, has not been reported to cause metabolic side effects, and is devoid of estrogenic or androgenic properties (Ibrahim *et al.* 2020, Raghupathy & Szekeres-Bartho 2022), and there is no convincing evidence of any causal association with fetal abnormalities; these attributes attest to the safety of the molecule (Katalinic *et al.* 2022). With respect to its use in threatened miscarriage, no significant difference has been reported compared to placebo in continuing pregnancy (Kuptarak & Phupong 2024). Use of oral dydrogesterone was preferred over vaginal progesterone in patients with vaginal bleeding during early pregnancy and a history of recurrent early pregnancy loss based on a numerically higher number of patients in the oral dydrogesterone group having successful continuation of pregnancy compared to the progesterone group (Kale *et al.* 2021).

The available evidence prompted us to investigate the efficacy of oral dydrogesterone versus oral micronized progesterone. As Devall *et al.* mentioned, there exists uncertainty over the effectiveness and safety of alternative progestogen treatments in threatened miscarriage (Devall *et al.* 2021). A pilot study pointed toward comparatively better symptom relief in patients treated for two weeks with oral dydrogesterone, although miscarriage rates were comparable between patients treated with oral dydrogesterone versus those treated with oral micronized progesterone (Siew *et al.* 2018), a finding that was corroborated by a study in which patients were treated till 12 weeks of gestation (Lou *et al.* 2021). The latter study also reported that the levels of sex hormones were significantly elevated and the incidence of adverse events (AEs) was significantly reduced in the dydrogesterone arm (Lou *et al.* 2021). Along similar lines, a comparatively better safety profile was reported in patients treated for two weeks with oral dydrogesterone compared to those treated with oral micronized progesterone (Shaikh *et al.* 2022). In a more recent study, resolution of pain and vaginal bleeding was better in patients treated till 12 weeks of gestation with oral dydrogesterone compared to those treated for similar duration with oral micronized progesterone (Verma & Yadav 2021). However, these studies comparing oral dydrogesterone versus oral micronized progesterone were single-center trials, in which patients were treated either for a predetermined period of two weeks or till 12 weeks of gestation.

The current study aims to combine the knowledge gained from the above studies and overcome some of their limitations. Accordingly, a multicentric, prospective clinical trial has been designed to compare oral dydrogesterone versus oral micronized progesterone in threatened miscarriage, wherein patients will be treated not for any predefined duration but till one week after stoppage of bleeding or if bleeding does not stop, then treatment will be continued till a maximum of 14 weeks of gestation (unless miscarriage is confirmed within 14 weeks of gestation or the investigator decides to prolong treatment for better outcome or if bleeding relapses). In addition to investigating miscarriage rates, symptom resolution, delivery outcomes and treatment-emergent AEs (TEAEs), this study aims to compare the immunomodulatory roles of the molecules being investigated by the assessment of cytokine levels as this aspect has not been explored in any of the aforementioned studies (Siew *et al.* 2018, Lou *et al.* 2021, Verma & Yadav 2021, Shaikh *et al.* 2022).

## Objectives

The primary objective of the study is to compare reduction in miscarriage rate before 20 weeks of gestation between the two treatment arms. The secondary objectives are to compare the following between the two treatment arms: rate of ongoing pregnancy at 24 weeks, rate of live births, safety of the study medications based on TEAEs, levels of immunomodulation markers and time to symptom resolution (Table 1).

**Table 1:** Study objectives.

**Primary objective**
• To compare the reduction in miscarriage rate before 20 weeks of gestation between pregnant women with threatened miscarriage treated with oral dydrogesterone versus oral micronized progesterone
**Secondary objectives**
• To compare the rates of ongoing pregnancy at 24 weeks and live births between women with threatened miscarriage treated with oral dydrogesterone versus oral micronized progesterone • To compare the safety of oral dydrogesterone versus oral micronized progesterone based on TEAEs in pregnant women with threatened miscarriage when treated with either • To compare the levels of immunomodulation markers, i.e., serum levels of cytokines (IL-4, IL-10, IFN-γ and TNF-α) in pregnant women with threatened miscarriage, before and after treatment with oral dydrogesterone versus oral micronized progesterone • To compare the time to symptom (vaginal bleeding and/or abdominal pain) resolution between women with threatened miscarriage treated with oral dydrogesterone versus oral micronized progesterone

TEAEs, treatment-emergent adverse events; IL, interleukin; IFN, interferon; TNF, tumor necrosis factor.

### Ethical considerations

The study is being conducted in accordance with the principles of Declaration of Helsinki, Good Clinical Practice (GCP) guidelines as per the International Council for Harmonization (ICH) of technical requirements for pharmaceuticals for human use, GCP guidelines issued by the Central Drugs Standard Control Organization (CDSCO), guidelines for clinical trials on pharmaceutical products in India as mentioned in New Drugs and Clinical Trials (NDCT) rules 2019 and guidelines set for clinical research by the Indian Council for Medical Research (ICMR), 2006, and Drugs and Cosmetic Rules, 1945. Patient recruitment was initiated only after obtaining written approval on the protocol from the ethics committee of each study site. Written informed consent will be obtained from every participant or her legally authorized representative before enrollment. Participants will be identified throughout and after study completion by allotted unique numbers; confidentiality will be maintained at all times.

### Trial registration

This trial has been registered with the Clinical Trials Registry-India (CTRI) on February 26, 2024 (reference number: CTRI/2024/02/063174).

### Study design and settings

This ongoing investigator-initiated study is a prospective, multicenter, open-label, randomized controlled interventional trial that will be conducted in 20- to 39-year-old pregnant women diagnosed with threatened miscarriage in their first trimester. The study is currently at the stage of patient screening and enrollment from multiple sites across India in order to ensure representation of investigators and patients from diverse populations. The study sites are as follows: Paras Hospitals (Gurugram, Haryana), IPGME&R and SSKM Hospital (Kolkata, West Bengal), Ramakrishna Medical Centre (Tiruchirappalli, Tamil Nadu), Dayanand Medical College & Hospital (Ludhiana, Punjab), Nowrosjee Wadia Maternity Hospital (Mumbai, Maharashtra), Manipal Hospital (Bengaluru, Karnataka), Sum Ultimate Medicare Hospital (Bhubaneswar, Odisha), Udyan Health Care Pvt. Ltd (Lucknow, Uttar Pradesh) and B. J. Medical College & Civil Hospital (Ahmedabad, Gujarat).

### Study endpoints

The study protocol outlines primary and secondary endpoints along with safety and exploratory endpoints, as mentioned in Table 2.

**Table 2:** Study endpoints.

**Primary endpoint**
• Miscarriage rate before 20 weeks of gestation
**Secondary endpoints**
• Ongoing pregnancy rate at 24 weeks • Change in serum levels of cytokines (IL-4, IL-10, IFN-γ and TNF-α) from baseline to the end of treatment • Time to symptom (vaginal bleeding and/or abdominal pain) resolution
**Safety endpoints**
• TEAEs • Changes in CBC • Change in results of LFTs from baseline to 14 and 24–26 weeks of gestation • Change in results of KFTs from baseline to 14 and 24–26 weeks of gestation
**Exploratory endpoints**
• Rate of live birth • Mode of delivery • Gestational age and birth weight of newborn • Resuscitation requirements • Congenital anomalies in the newborn • History of pre-eclampsia

IL, interleukin; IFN, interferon; TNF, tumor necrosis factor; TEAEs, treatment-emergent adverse events; CBC, complete blood count; LFTs, liver function tests; KFTs, kidney function tests.

### Eligibility criteria

Consenting participants will be enrolled if they satisfy all inclusion criteria and none of the exclusion criteria. The eligibility criteria are listed in Table 3.

**Table 3:** Eligibility criteria.

**Inclusion criteria**
• Pregnant women aged 20–39 years with threatened miscarriage (vaginal bleeding and/or abdominal pain) in the first trimester • Gestational age between 5 and 12 weeks • Euthyroidism or controlled hypothyroidism (based on medical history) • Presence of a viable pregnancy • Presence of intrauterine gestational sac on USG if a urine pregnancy test is first positive within the past two weeks • BMI ≥18 and ≤30 kg/m^2^ • Willingness to provide written informed consent
**Exclusion criteria**
• Pregnant women with inevitable abortion • History of recurrent miscarriage defined as at least two consecutive spontaneous miscarriages • Heavy vaginal bleeding or severe abdominal pain requiring surgical intervention • Presence of intrauterine fetus with a crown-rump length inappropriate for gestational age, with no visible heartbeat, or a mean gestational sac of ≥25 mm with no visible fetal pole on USG • Evidence of ectopic pregnancy • Conceived on gonadotrophins or with the use of assisted reproductive technologies • Abnormalities in the structure of the uterus or amputation of the cervix, or any other genital tract anomalies • Uterine myoma with submucosal location of the node (a clinically significant size as judged by the investigator) • Anembrion or fetal malformations as established causes of loss of previous pregnancies • Other clinically significant causes of miscarriage identified during examination (including but not limited to prepregnancy diabetes, prepregnancy uncompensated thyroid dysfunction, history of malignant tumors or current tumors or psychiatric illnesses) • Known STDs • Administration of enzyme-inducing medicinal products (anticonvulsants, antipsychotics, antidepressants and tranquilizers) or use of psychoactive substances before and during pregnancy • Multiple pregnancy • Known as having an endocervical polyp • Known as having infection such as pneumonia, pyelonephritis or septicemia • Known as having autoimmune diseases such as systemic lupus erythematosus, systemic sclerosis or rheumatoid arthritis • Known as having a coagulation defect • Known as having severe heart, liver, lung, kidney or any other organ disorder • Current or ongoing substance abuse, including alcohol and tobacco, as determined by the investigator • History of chemotherapy or radiotherapy • Known allergy or hypersensitivity to dydrogesterone or oral micronized progesterone • Use of hCG or dydrogesterone or progesterone within one month before study enrollment • Participation in any other clinical trial within 30 days before the study enrollment • Planned participation in any other trial during the entire duration of the study • Refusal or inability to comply with the requirements of the protocol for any reason, including scheduled clinic/hospital visits and laboratory tests • Any other condition(s) which would make the patient, in the opinion of the investigator, unsuitable for the study • Any other clinical condition(s) that, as judged by the investigator, contradict(s) inclusion criteria, may lead to early termination of the subject’s participation in the study or make it difficult to interpret the results obtained in the study

BMI, body mass index; hCG, human chorionic gonadotrophin; USG, ultrasonography; STDs, sexually transmitted diseases.

### Sample size

A total of 302 subjects are required to detect a difference of 5% (Siew *et al.* 2018) in miscarriage rate between the two treatment arms, with a confidence level of 95%, power of 80% and noninferiority margin of 15%, taking into account an anticipated dropout rate of 8%. The enrolled subjects will be assigned equally to the four strata (as described below) within each treatment arm; therefore, the sample size has been selected as 304. Thus, 152 subjects would be required in each treatment arm at baseline.

### Randomization

All enrolled subjects will be randomized in a 1:1 allocation ratio to receive either oral dydrogesterone or oral micronized progesterone. A computer-generated central randomization list will be divided among the study sites. Randomization will ensure that there is equal participation of subjects aged ≤30 and >30 years, and equal participation of subjects with body mass index (BMI) ≤25 and >25 kg/m^2^ within each treatment arm. To attain this, stratified randomization technique will be used. There will be four strata within each treatment arm: (a) age ≤30 years, BMI ≤25 kg/m^2^; (b) age ≤30 years, BMI >25 kg/m^2^; (c) age >30 years, BMI ≤25 kg/m^2^; and (d) age >30 years, BMI >25 kg/m^2^.

### Treatment

Subjects in the test arm will be treated with oral dydrogesterone; the first dose will be 40 mg (stat) and subsequent doses will be 10 mg three times a day. Subjects in the reference arm will be treated with oral micronized progesterone at a dosage of 200 mg two times a day. Except for subjects who face miscarriage within 14 weeks of gestation, rest will be treated up to one week after stoppage of bleeding or if bleeding does not stop, then treatment will be continued till a maximum of 14 weeks of gestation. Continuation of treatment beyond this will solely be at the discretion of the treating physician. If relapse of bleeding is observed after stoppage of treatment, the physician may decide to continue the medication at his/her discretion; relevant details will be recorded.

### Schedule of visits

**Figure 1:**
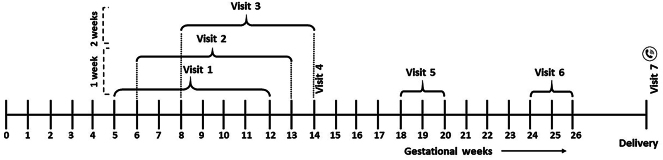
Schedule of visits. All study subjects will be screened and enrolled at Visit 1 (at 5–12 weeks of gestation) after obtaining written informed consent. Visit 2 will be conducted between 6 and 13 weeks and Visit 3 between 8 and 14 weeks of gestation. Visit 4 will be conducted at the end of 14 weeks of gestation, with a permissible window period of +3 days. Visits 5 and 6 will be conducted at 18–20 and 24–26 weeks of gestation, respectively. Visit 7 will be conducted after parturition.

A total of seven visits are planned in this study: three in the first trimester, three in the second trimester and one after delivery. The schedule of visits is represented graphically in Fig. 1. Treatment will be initiated at Visit 1 and completed at Visit 4 (except for subjects with prolonged treatment based on physician’s discretion); a window period of +3 days is permissible for Visit 4. Visit 7 will be conducted after delivery. In addition to these scheduled visits, subjects may consult the investigator for any emergent situation during study participation.

### Planned evaluations

**Figure 2:**
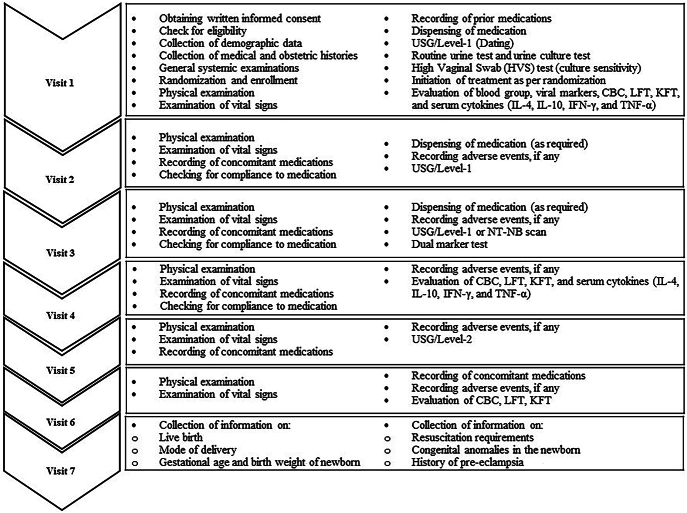
Study flowchart. There will be a total of seven visits in this study. A detailed list of activities to be performed at each visit is presented here.

Subjects will be screened and enrolled after obtaining written informed consent at Visit 1. Routine pregnancy-specific imaging and laboratory investigations will be done at a central laboratory during study participation. Subjects will be monitored throughout participation for AEs and serious AEs. Activities planned at each study visit are detailed in Fig. 2.

### Data collection and management

Data from source documents will be transcribed in English to case report forms (CRFs) either by the investigator or by trained and delegated clinical research coordinators; confidential details of subjects will be redacted. Accuracy, completeness and legibility of data will be ensured and then shared by the investigator in a timely manner to all those concerned. Edit checks will be run; queries will be resolved as per in-house standard operating procedures (SOPs) of the assigned Clinical Research Organization (CRO) and clarification/correction will be obtained from the investigator through data clarification forms (DCFs) in case of missing data, transcription error or new information. This will be followed by review and if data are found accurate, the database will be locked. Subsequently, data will be extracted for statistical analysis as per in-house SOPs. Relevant study documents, along with unused or spurious data, will be kept in due confidentiality for at least five years from study completion, after which these will be destroyed or retained further, as appropriate.

### Study monitoring

Monitoring will be done as per in-house SOPs of the assigned CRO to comply with the aforementioned ethical guidelines. Monitoring will include verification of subject recruitment as per eligibility criteria, source documentation, completeness and accuracy of CRFs and specified trial-related responsibilities of the investigator and trial staff, and ensure that there is no participation of unauthorized individuals in the study conduct. Adequate and accurate documents will include site master file (protocol; sample of CRF, ICF and DCF; notification, submission and approval letter of the ethics committee; staff curriculum vitae; staff job authorization forms etc.) and clinical source documents of subjects. Site participation may be terminated if noncompliance is noted. Formal site closure will be done after all study-related activities have been completed; ethics committees will be notified by the respective investigators.

### Quality assurance

Auditing will be performed as per in-house SOPs of the assigned CRO to ensure study compliance with the aforementioned ethical guidelines and to maintain the quality and integrity of data. The auditor shall be given access to all study-related documents. To ensure confidentiality, patient identification information will be concealed from copies of medical documents made for audit purposes. Findings of the auditor will be documented and informed to the study site; prompt and necessary action will be taken in case of noncompliance.

### Statistical methods

All data analysis will be carried out as per comprehensive statistical analysis plan and based on the ICH E9 document ‘Statistical Principles for Clinical Trials’. All statistical analyses will be done using the Statistical Package for Social Sciences (SPSS; IBM Corp., USA). Continuous variables will be checked for normality and analyzed using paired t-test if the distribution is normal, or Wilcoxon signed rank test otherwise. Descriptive statistics and proportions will be used to summarize the population in each treatment arm/subgroup. Categorical variables will be analyzed using McNemar’s test or Chi-square test, as applicable. Regression analysis will be used to analyze the association between variables. Incidence of AEs will be reported as the proportion of subjects reporting them.

## Discussion

Both oral dydrogesterone and oral micronized progesterone have been successfully used to treat pregnant women with threatened miscarriage (Siew *et al.* 2018, Lou *et al.* 2021, Verma & Yadav 2021, Shaikh *et al.* 2022). This study aims to add information to the existing literature on the contribution of the study medications to change in levels of cytokines (such as IL-4, IL-10, IFN-γ and TNF-α) during pregnancy.

Dydrogesterone has been shown to inhibit the synthesis of IFN-γ and TNF-α and upregulate the synthesis of IL-4 and IL-6, thus inducing a Th1 to Th2 cytokine shift (Raghupathy *et al.* 2005). The increase in IL-10 and decrease in IFN-γ concentrations upon dydrogesterone treatment have been demonstrated by Hudić *et al.* (2011). In a more recent clinical trial, albeit conducted in a small population of pregnant women with threatened miscarriage, dydrogesterone resulted in a significant decrease in Th1/Th2 ratio and 93.8% patients had continued pregnancies (Ibrahim *et al.* 2020).

Taken together, there is evidence supporting the clinical efficacy, safety and immunomodulatory functions of progestogens. Accordingly, this ongoing study is being conducted to inform on the comparative efficacy and safety of oral dydrogesterone versus oral micronized progesterone in threatened miscarriage. In addition to the assessment of rates of miscarriage, rates of successful continuation/completion of pregnancy, time to symptom resolution, outcomes at delivery and incidence of TEAEs, treatment-induced changes in cytokine levels will be evaluated along with the routine investigation of other blood-related parameters such as complete blood count (CBC), liver function tests (LFTs) and kidney function tests (KFTs). This study is thus expected to elucidate multiple aspects of progestogen administration and add worthwhile data to the currently available information on their usage in threatened miscarriage.

## Declaration of interest

The authors declare that there is no conflict of interest that could be perceived as prejudicing the impartiality of the ongoing work.

## Funding

Funding for this study was provided by Mankind Pharma Limited. The authors are solely responsible for the final content of the manuscript.

## Author contribution statement

AK is the principal investigator of this investigator-initiated study. GSK, TRD, AT, AP, GKN and TP are coinvestigators who, along with AK, were involved in protocol preparation. AK, GSK, TRD, AT, AP, GKN, TKP, TG and MJ will participate in patient recruitment, treatment and data collection. MM was involved in protocol and manuscript preparation. The manuscript has been read and approved by all authors. The order of authors is independent of their individual contributions to the study.
